# Treatment of Noninfectious Retinal Vasculitis Using Subcutaneous Repository Corticotropin Injection

**DOI:** 10.18502/jovr.v16i2.9086

**Published:** 2021-04-29

**Authors:** Stephen D. Anesi, Peter Y. Chang, Arash Maleki, Andrew Stephenson, Alyssa Montieth, Artur Filipowicz, Sarah Syeda, Soheila Asgari, Marisa Walsh, Jamie Lynne Metzinger, C. Stephen Foster

**Affiliations:** ^1^Massachusetts Eye Research and Surgery Institution, Waltham, MA, United States; ^2^The Ocular Immunology and Uveitis Foundation, Waltham, MA, United States; ^3^Noor Ophthalmology Research Center, Noor Eye Hospital, Tehran, Iran; ^4^Harvard Medical School, Department of Ophthalmology, Boston, MA, United States

**Keywords:** Acthar, Corticotropin Gel, Fluorescein Angiography, Ocular Inflammation, Retinal Vasculitis, Uveitis

## Abstract

**Purpose:**

To show whether subcutaneous repository corticotropin injection (RCI, Acthar® Gel, a repository corticotropin injection, can be an effective potential therapeutic agent for noninfectious retinal vasculitis.

**Methods:**

Patients with active retinal vasculitis were followed with serial ultra-wide-field fluorescein angiograms and treated with 80 units of subcutaneous repository corticotropin injection twice weekly.

**Results:**

Primary outcome of ≥50% improvement in response level (RL) for retinal vasculitis and percent improvement in retinal vasculitis severity scoring (RVSS) by more than one quartile (≥25%) at week 12 was met in 15 and 16 of the 30 total eyes, respectively, including 1 eye with severe retinal vasculitis in each group. Complete resolution of retinal vasculitis was seen in seven eyes with a mean time of 17.1 weeks. Intraocular pressure elevation requiring therapy and cataract progression were noted in two and three eyes, respectively. One patient stopped medication due to side effects (injection site reaction).

**Conclusion:**

Repository corticotropin injection was well-tolerated overall. Repository corticotropin injection may be an effective therapeutic agent in the treatment of noninfectious retinal vasculitis.

##  INTRODUCTION

Retinal vasculitis (RV) represents a group of sight-threatening and typically stubborn forms of ocular inflammation which may affect retinal arteries, veins, or capillaries. It can be potentially detrimental to vision by way of occlusive or nonocclusive mechanisms, leading to secondary unilateral or bilateral findings of retinal ischemia, macular edema, neovascular changes, retinal detachment, vitreous hemorrhage, and secondary glaucoma.^[1,2]^ Noninfectious RV may present alone or as a manifestation of or an association with a wide variety of other ocular inflammatory diseases like idiopathic intermediate, posterior, and panuveitis or even potentially severe systemic conditions such as sarcoidosis and Adamantiades-Behçet's disease.^[1,3]^

The anatomy of the eye is such that examination allows for direct visualization of retinal vasculature, allowing one to observe and perform imaging of *in vivo* vascular structures during the course of active disease as well as response to employed therapeutic measures. Findings suggesting RV include directly visible measures such as retinal sheathing, overlying inflammatory exudates, segmental vascular narrowing, as well as secondary signals of ischemia such as cotton wool spots and intraretinal hemorrhages. Sometimes, however, this inflammation is not detectable via ophthalmoscopy alone. Fluorescein angiography (FA) has long been the gold standard for imaging and evaluation of RV and other related entities. The availability of ultra-wide-field fluorescein angiography (UWFA) now allows greatly improved visibility and recognition of pathology involving the retina and retinal vasculature, particularly in the peripheral retina, and may also allow for imaging through a small pupillary aperture.^[4,5]^

Recommended therapeutic options for sight-threatening noninfectious uveitis, as well as RV, generally include anti-inflammatory regimens involving corticosteroids in the short term followed by steroid-sparing therapy utilizing immunosuppressive medications, including chemotherapeutic (disease-modifying anti-rheumatic drugs or DMARDs) or biologic agents.^[2,6,7,8]^ Novel means of addressing severe or recalcitrant ocular inflammation have been studied to give providers more options to choose from when trying to reduce or eradicate inflammation without relying on systemic corticosteroids.

Target molecules of more recently renewed interest include melanocortin peptides such as adrenocorticotropic hormone (ACTH), a key part of the signaling involved in the hypothalamic–pituitary–adrenal axis, and its cleavage product alpha-melanocyte stimulating hormone (α-MSH). ACTH is thought to help suppress inflammation in part by a “steroid-dependent” mechanism by causing upregulation of the endogenous glucocorticoid in the adrenal cortices.^[9,10]^ Other melanocortin, such as α-MSH, bind to and activate separate melanocortin receptors (MCRs) from ACTH, and have also been shown to be instrumental in immune regulation in ocular inflammatory diseases via a separate “steroid-independent” mechanism.^[11,12]^ α-MSH has long been known to contribute to ocular immune regulatory mechanisms and immune privilege.^[13]^ *In vitro* studies have shown that α-MSH may suppress both the innate and adaptive immune response, and can induce the conversion of effector T-lymphocytes into regulatory T-cells (Tregs) which both suppress interferon-gamma (IFN-γ) production and produce transforming growth factor-beta (TGF-β).^[14,15]^ There are five known human MCRs, one of which (MCR2) exclusively binds ACTH and is responsible for cortisol production. The other MCRs (MCR1, MCR3-5) strongly bind α-MSH (and to a lesser degree β- and γ-MSH) in various cells throughout the body including several located in ocular tissues, notably retina and retinal pigment epithelium, as well as various leukocytes involved in both innate and adaptive immunity.^[16]^

Repository corticotropin injection (RCI, Acthar® Gel) is a naturally sourced complex mixture of adrenocorticotropic hormone analogs and other pituitary peptides. A major component in the formulated complex mixture is N-25 deamidated porcine ACTH (1-39). Per the label, the major component is N-25 deamidated ACTH. If needed, you may further expand the description with the following: “The Acthar Gel manufacturing process converts the initial porcine pituitary extract with low ACTH content into a mixture having modified porcine ACTH and other related peptide analogs solubilized in gelatin. A major component in the formulated complex mixture is N-25 deamidated porcine ACTH (1-39). RCI is known to bind to and activate all five MCRs in humans. It has an indication for and has been used to treat several systemic immune-mediated diseases including rheumatoid arthritis, systemic lupus erythematosus, sarcoidosis, and multiple sclerosis, and also has an FDA indication for treating severe acute and chronic allergic and inflammatory processes involving the eye and its adnexa. Because of its unique mechanism, RCI represents a novel approach to treating ocular inflammatory disease, including disease that may be refractory to other forms of therapy. More recent data, unfortunately, on the use of RCI in treating ocular inflammation is currently limited only to case reports.

The purpose of this study is to further evaluate the possible effectiveness of RCI in treating patients with retinal vasculitis who may either be treatment naïve or who have failed other forms of therapy. It is the hypothesis of the authors that use of RCI in these patients may lead to improvement in markers of inflammatory activity on examination and imaging, and potentially to resolution of active inflammation altogether.

##  METHODS

### Study Setting and Patient Population 

This was a prospective, nonrandomized, open-label, proof-of-concept study conducted at a single, tertiary care uveitis center. Patients with active retinal vasculitis as a manifestation of noninfectious ocular inflammatory disease were identified during routine follow-up care and invited to participate. The inclusion criteria for the study were: adult patients aged ≥18 years with active retinal vasculitis (involving arteries, capillaries, or veins) with a visible fundus via wide-field FA in the study eye and willingness to participate. The exclusion criteria included: patients who were currently pregnant; active infectious ocular, extraocular, and/or systemic disease; history of malignancy (with the exception of dermatologic entities of basal or squamous cell carcinoma which have been excised); systemic illness involving abnormalities of the hypothalamic–pituitary–adrenal axis; primary adrenocortical insufficiency or adrenocortical hyperfunction; known hypersensitivity to study drug and/or diagnostic tools; other severe disease that warrants critical attention and renders patients unable to participate in the study; retinal disease that may confound or obfuscate findings of retinal vasculitis (i.e., retinal vascular occlusion, significant diabetic retinopathy), and no visible fundus on dilated fundoscopy. Patients were not enrolled if they had been treated with any oral, periocular, or intraocular suspension of corticosteroid within six weeks prior to the study; any intraocular dexamethasone corticosteroid implant within six months prior to the study; or any long-lasting fluocinolone corticosteroid implant within three years prior to the study. Additionally, patients already prescribed and adherent to non-steroidal immunosuppressive therapy continued their regimens throughout the study, but no modification of dose (increase or decrease) was permitted within six weeks of initiating the trial and throughout the duration of the treatment and follow-up periods. Exceptions for changes in concomitant medications were only made for any patient that was deemed in need of rescue therapy by the investigator at any time during the study because of continued significant active disease despite study medication use, or if there was deemed poor tolerance of the medication and the patient required additional therapy to help control inflammation – these patients were then excluded from further analysis of clinical response of ocular inflammation to RCI.

Safety data were extracted from all patients who had at least one follow-up visit after starting medication. The per protocol population (PPP) was defined by eyes of patients who were able to participate in the study without deviation from protocol for any portion of the study after the initial visit, and data extracted from the PPP was only utilized from visits for which strict adherence to the study protocol was maintained without deviation. Deviations from study protocol included introduction of additional anti-inflammatory or immunomodulatory therapy, stoppage of the study medication, or any other reason for which the participant was no longer deemed appropriate by the investigator to include in the study prior to study completion.

Institutional Review Board approval was obtained prior to the initiation of the study. The study was conducted in accordance with the Declaration of Helsinki and adhered to Good Clinical Practice guidelines. The clinical trial was registered and can be found at ClinicalTrials.gov identifier: NCT03066869. Each subject signed an informed consent before participating in the study.

### Definition of Response Level 

Response level (RL) was defined at each visit as predefined percentage changes in retinal vasculitis activity versus baseline activity at the initial visit, with positive RL values indicating a reduced level of activity or a positive response while on therapy, and negative RL values indicating an increased level of activity or a negative response while on therapy. Active retinal vasculitis status was defined as angiographic leakage on wide-field FA from retinal arteries, capillaries, or veins, as determined by one of the three investigators at the time of baseline and subsequent visits. RL scores used in data collection were determined after study completion by two of the investigators (SDA and PYC) independently assessing imaging without referencing other clinical data from the visits. RLs were graded in steps of either 0, 12.5 (1–12.5), 25 (13–25), 50 (26–50), 75 (51–75), or 100 (76–100) percent for improvement; if worsening occurred, the same was done with a negative score given. Scores were later compared for possible discrepancy in grading, and if any were found, an acceptable score was then agreed upon by both investigators by utilizing the historical clinical impression of the investigator at the time of each visit.

### Retinal Vasculitis Severity Scoring

To describe RV severity, retinal vasculitis severity scoring (RVSS) was performed at the baseline, week-12, and week-24 visits. The scoring system was based on five sections, including an area 2.5 to 3 disc diameters surrounding the fovea incorporating the temporal arcades designated as the macula, and then diagonal separation of the superior, nasal, inferior, and temporal peripheral quadrants outside the arcades. Each section received a score of 0 to 3 based on the severity of leakage in the late angiographic phase (venous phase). Macular scoring was based on small vessel or capillary leakage in macular quadrants: 0 for no leakage, 1 for one quadrant, 2 for two to three quadrants, and 3 for all four quadrants. Peripheral quadrant leakage was scored thusly: 0 for no leakage, 1 for staining of vessels with minimal leakage, 2 for more leakage with distinct vascular margins, and 3 for more leakage obscuring the vessel margins. Total score initially of 1 to 15 was possible, and scoring was tabulated with retinal vasculitis severity score given as thus: mild for score 1 to 5, moderate for 5.5 to 10, and severe for 10.5 to 15. This scoring was applied by two investigators (SDA and PYC) independently assessing imaging without referencing other clinical data from the visits separately from the assessment of RL. These values were then averaged for a final score at each time point. Baseline-visit measurements were used in the initial description of retinal vasculitis severity.

For comparison to the RL scoring previously described, the week 12 and week 24 RVSS were individually divided by the baseline RVSS, and these numbers were then subtracted from 1 and multiplied by 100 to give an RVSS percent improvement (or worsening if negative) in each subject from baseline. Of note, no worsening above a score of 15 was possible, limiting this method in this direction, however, this problem was not encountered.

### Data Collection

Patients were seen at baseline, as well as subsequent visits occurring at weeks 2, 4, 8, 12, and 24 after initiation of RCI, with unscheduled visits permissible if necessary. Study medication for all patients was started within two weeks following the baseline visit. Data collected included best-corrected visual acuity (BCVA), intraocular pressure, slit lamp biomicroscopy findings, dilated fundus examination, wide-field FA, weight, vital signs (blood pressure and heart rate), and laboratory assessments. Macular imaging via spectral domain optical coherence tomography (OCT) was obtained per investigator initiative. Biomicroscopic findings included anterior chamber (AC) cell and flare as well as vitreous cell and haze as scored per SUN criteria.^[17]^ All assessments were repeated at each visit after baseline, apart from laboratory assessments, which were carried out at weeks 4, 12, and 24 only to assess for potential toxicity to medication.

WFA was performed for the study eye. Fundus photography, red-free photographs, and fundus autofluorescence were all completed prior to intravenous injection of fluorescein dye. Photographs were taken immediately at dye injection, then every 10 s in the study eye followed by alternate eye, for up to 30 s. Subsequently, photographs were captured every 30 s in the study eye, followed by the fellow eye for up to 2 min following the dye injection, then once per minute in the study eye, followed by the fellow eye for up to 7 min following the dye injection. OCT was performed at initial assessment visit for most patients and then as needed; central macular thickness (CMT) was reported for patients if available.

### Study Outcome Measures

The primary outcome measure was the percentage of PPP eyes that improved during the study: (1) eyes that had ≥50% improvement by definition in RL by the week-12 visit or (2) eyes with RVSS that improved by more than one quartile (>25%) by week 12.

Secondary outcomes included numeric RVSS at baseline, week 12, and week 24. Other secondary outcomes were vision, intraocular pressure, AC cell and flare, vitreous cell and haze, weight, blood pressure, and heart rate. CMT was collected via OCT for most patients, and follow-up OCT was obtained for patients with cystoid macular edema, which was defined as baseline CMT of 300 or more or demonstrable intraretinal fluid.

Safety assessments, including drug tolerability, adverse events, and all other ocular and/or systemic complications were also evaluated. Side effects of RCI and reasons for therapy discontinuation were gathered.

### Treatment Protocol

RCI was initiated at 80 units given subcutaneously twice weekly after the baseline visit. Any changes made in the treatment regimen during the study resulted in the patient and eye(s) to no longer be included in the PPP at any subsequent visits. Patients who did not show clinical or objective evidence on the assessment of improvement while taking therapy were rescued at 12 weeks; patients who showed worsening or concern for their condition prior to 12 weeks were also rescued with appropriate therapy at the discretion of the investigator. Participants that were rescued were also followed through 24 weeks.

### Statistical Analysis

Categorical variables were described as counts and percentages. Continuous variables were described as means, standard deviations, and ranges. Statistical analysis was performed with R Statistical Package (version: 3.5.2). Categorical variables were compared at each follow-up visit with the one before except the baseline and two weeks using Chi-square and Fisher's exact test. Q-Q plot was used to examine the normal distribution of quantitative variables. Linear random mixed model was used to show the 24-week trends. In the analyses, the correlation between two eyes and the follow-up times (12- and 24 week) were examined in an unstructured correlation matrix. The repeated measures analysis of variance was used to determine 24-week change of weight, HR, systolic and diastolic pressures. The eye was the unit of all calculations except for demographics and systemic side effects, where the patient was the unit of calculations. *P*-value ≤ 0.05 was considered significant.

##  RESULTS

Twenty patients were originally recruited for the study. One patient did not return after the first visit and was thus excluded from any analysis. Another patient (one eye) was excluded because of shallow peripheral retinal detachment. Thirty-one eyes in 19 patients were followed until at least the second visit. Table 1 lists a summary of patients' demographic information.

**Table 1:** Demographics – characteristics and baseline details

**Patient demographics**	
Patients enrolled	20
Patients with ≥1 follow-up (patients [eyes])	19 [31]
Patients followed per protocol (patients [eyes])	18 [30]
Mean age (yr)	43.0
Sex, *n* (%)	
Female	17 (89.5)
Male	2 (10.5)
Race, *n* (%)	
Caucasian	16 (84.2)
Hispanic or Latin	2 (10.5)
South Asian	1 (5.3)
Patients with uveitis, *n* (%)	18 (94.7)
Uveitis diagnosis, *n* (%)	
Anterior	2 (10.5)
Intermediate	3 (15.8)
Posterior	1 (5.3)
Panuveitis	12 (63.2)
Mean time with uveitis prior to the study (yr)	5.8± 3.93
Mean time with active symptoms prior to the study (months)	2.0± 2.31
Patients with idiopathic disease, *n* (%)	9 (47.4)
Patients with systemic association, *n* (%)	10 (52.7)
HLA-B27	3 (15.8)
Multiple sclerosis	2 (10.5)
Sarcoidosis	5 (26.3)
Patients on systemic anti-inflammatory medication, *n* (%)	8 (42.1)

**Table 2:** Comorbidities and complications of inflammation

**Complications**	**Number (%)**
Cataract	10 (52.6%)
Pseudophakia	3 (15.8%)
Glaucoma/ocular hypertension	6 (31.6%)
Cystoid macular edema	6 (31.6%)
Epiretinal membrane	2 (10.5%)
Peripheral retinal ischemia	1 (5.3%)
Papillitis	3 (15.8%)

**Table 3:** Characteristics of retinal vasculitis and uveitis

**Characteristics of retinal vasculitis eyes (** ***n*** ** = 31)**	**Total**
Vasculature involvement, *n* (%)	
Veins	31 (100)
Capillaries	19 (63.2)
Arteries	1 (3.2)
retinal vasculitis (RV), *n* (%)	
Mild	15 (48.4)
Moderate	10 (32.3)
Severe	6 (19.4)
Quadrants involved, *n* (%)	
1	4 (12.9)
2	6 (19.4)
3	8 (25.8)
4	13 (42.9)
Mean number of quadrants involved	2.97
Types of uveitis	Total patients (*n* = 19)
Anterior uveitis	2 (10.5)
Intermediate uveitis	1 (5.3)
Posterior uveitis	2 (10.5)
Panuveitis	3 (15.8)
RV, retinal vasculitis This was changed to retinal vasculitis (RV)	

**Table 4:** Changes in retinal vasculitis scoring system (RVSS) and response level (RL) from baseline to week 12 and week 24. *P*-value was < 0.001 for the 24-week change of study indices by repeated measures analysis of variance without applying correlation between fellow eyes, as well as comparison of baseline and week 12, baseline and week 24, and weeks 12 and 24 with applying correlation between fellow eyes.


	**Baseline**	**Week 12**	**Week 24**
RVSS${}^{1}$	6.92 ± 3.83	5.19 ± 4.67	3.81 ± 4.68
RL${}^{2}$ (OS)${}^{3}$ (%)	0.00 ± 0.00	37.72 ± 31.50	61.06 ± 34.76
RL${}^{2}$ (SS)${}^{4}$ (%)	0.00 ± 0.00	59.82 ± 34.73	74.11 ± 34.13
${}^{1}$Retinal vasculitis scoring system; ${}^{2}$Response level; ${}^{3}$Objective scoring; ${}^{4}$Subjective scoring			

**Table 5:** Other ocular parameters


	**Screening**	**12 weeks**	**24 weeks**	**** ***P*** -value*
BCVA (logMAR)	0.28 ± 0.22	0.22 ± 0.22	0.18 ± 0.22	**<0.001**
IOP (mmHg)	14.36 ± 4.48	14.64 ± 4.09	13.64 ± 4.95	**<0.001**
AC cell	0.57 ± 0.92	0.00 ± 0.00	0.07 ± 0.18	0.264
AC flare	0.00 ± 0.00	0.36 ± 0.74	0.14 ± 0.36	ND
Vitreous cell	0.04 ± 0.13	0.14 ± 0.23	0.11 ± 0.21	**<0.001**
Vitreous haze	0.25 ± 0.67	0.00 ± 0.00	0.29 ± 0.47	0.998
BCVA, best-corrected visual acuity; IOP, intraocular pressure; AC, anterior chamber; LogMAR, logarithm of the minimum angle of resolution				

The mean length of time with known diagnosis of retinal vasculitis prior to screening was 5.8 ± 3.93 years (range, 0 to 13). Then mean time since the onset of symptoms for current active inflammation was 2.0 ± 2.31 months (range, 0 to 7); 9 of 19 patients (47.4%) had idiopathic disease and 10 had an associated systemic disorder; 3 (15.8%) had HLA-B27-associated uveitis, 2 (10.5%) had multiple sclerosis, and 5 (26.3%) had sarcoidosis [Table 1]. At the time of screening, 8 of the 19 patients (42.1%) were already on some form of systemic anti-inflammatory or immunosuppressive therapy – these medications included mycophenolate mofetil, cyclosporine, interferon beta-1a, methotrexate, adalimumab, infliximab, sulfasalazine, and ibuprofen.

Table 2 demonstrates the comorbidities and/or complications of inflammation in 19 patients at the time of screening.

**Figure 1:**
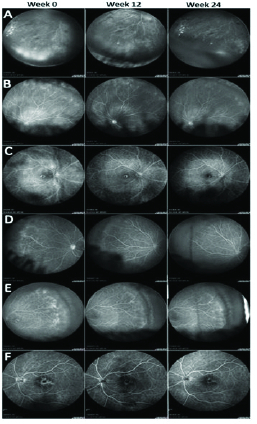
Fluorescein angiography of per protocol study eyes (A–F) demonstrating changes in retinal vasculitis and generalized inflammation from week 0 (baseline) to week 12 to week 24. Of note, varying levels of improvement were seen, from mild to moderate to complete resolution by week 24.

Table 2 presents the uveitis and characteristics of retinal vasculitis. Examples of responses of RV in various PPP eyes at weeks 12 and 24 are shown in Figure 1.

**Figure 2:**
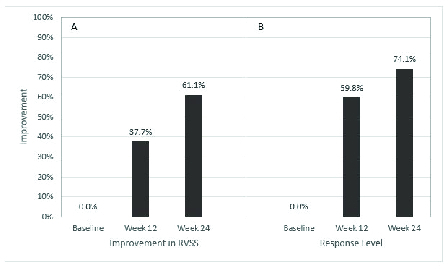
Comparison of response level (RL) between objective retinal vasculitis scoring system (A) and subjective retinal vasculitis scoring system (B).

**Figure 3:**
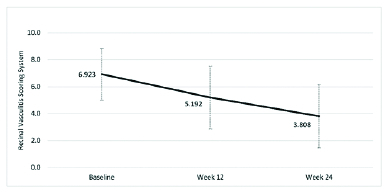
Mean RL for PPP eyes at each study visit after baseline.

**Figure 4:**
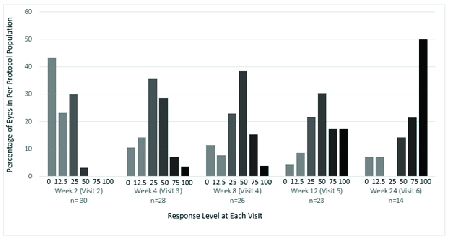
Percentage of PPP eyes with varying RL at each study visit after baseline.

**Figure 5:**
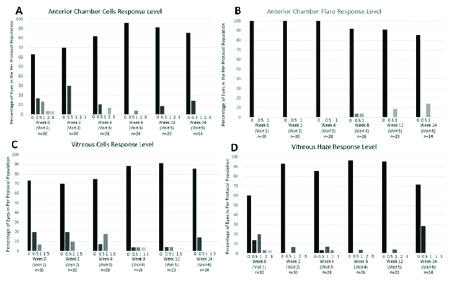
Slit lamp characteristics showing (A) anterior chamber (AC) cells, (B) AC flare, (C) vitreous cells, and (D) vitreous haze at each study visit.

Furthermore, 30 eyes of 18 patients with active RV were included in the PPP. Fourteen of the eighteen patients (23 of the 30 eyes [76.7%]) were able to continue RCI in the PPP through 12 weeks. Nine of the eighteen patients (14 of the 30 eyes [46.7%]) were then able to continue RCI through the entirety of the 24 weeks of the study without change in protocol. Nine eyes (30.0%) did not continue in the PPP past week 12 – three patients (five eyes) due to ineffectiveness, one (two eyes) due to noncompliance, and one (two eyes) due to side effects as well as needing additional treatment.

### Therapeutic Response – Retinal Vasculitis

The primary outcome of improvement in RL of at least 50% or more [Figure 2], or improvement in RVSS to at least the second quartile or more at week 12 [Figure 3] was seen in 15 and 16 of 30 total eyes, respectively (50.0% and 53.3%), and of the 23 eyes that were followed on the study protocol without deviation to week 12, respectively (65.2% and 69.6%). Changes in RVSS, % improvement via RVSS, and RL between baseline and week 12, between weeks 12 and 24, and between baseline and week 24 were statistically significant (*p* < 0.001) [Table 4]. Of the 15 who improved in RL to 50% by week 12, 7 were classified as having mild RV, 7 moderate, and 1 severe. Of the 16 who improved in RVSS to the second quartile or more by week 12, 8 were classified as having mild, 7 moderate, and 1 severe RV.

Of all 30 PPP eyes, the number of eyes followed in the study without deviation in protocol included 30 at week 2, 28 at week 4, 26 at week 8, 23 at week 12, and 14 at week 24. The mean RL and RVSS along with percent improvement in RVSS for PPP eyes at baseline, weeks 12 and 24 are shown in Figure 4. Time to any improvement and time to 50% improvement in RL in the total PPP eyes are demonstrated in Figure 5.

Moreover, 7 of the 30 eyes (23.3%; CI 12.3 to 45.9%) experienced complete resolution of disease by RL at the end of RCI use. The mean time to resolution was 17.1 weeks (range 12 to 24).

### Therapeutic Response – Other Ocular Parameters

Changes in the mean BCVA and intraocular pressure for all PPP eyes from screening to 12 weeks, and to 24 weeks were statistically significant (*P* < 0.001) [Table 5]. Percentages of PPP eyes for measures of anterior and posterior uveitis at each visit are shown in Figure 6.

OCT evaluation at screening occurred in 23 eyes of 14 patients. CME was found in eight eyes of six patients. Improvement in CME was seen in five eyes (62.5%) of four patients during the PPP visits, with complete resolution occurring in three eyes (37.5%) of three patients during the PPP visits.

### Safety Measurements

Thirteen of the eighteen PPP (21 eyes) used RCI throughout the entire 24-week course of the study. One patient stopped RCI during the study due to an adverse reaction (injection site reaction), one due to compliance, and three due to poor efficacy of medication. Nine of the thirteen patients to complete the course (14 eyes) had no change in therapy through the entire study, while the other four of the thirteen received additional systemic or local therapy to help control inflammation. Other nonserious adverse events felt to be attributable to the addition of RCI to therapy in this study group were insomnia (two patients), mood change (one patient), loss of appetite (one patient), and dizziness with mild nausea (one patient). No concerning changes in laboratory values were found in any patient during the study.

The mean weight in PPP eyes at screening, week 12, and week 24 was 184.16 ± 48.83, 184.52 ± 49.90, and 189.31 ± 48.47 pounds, respectively (*p* < 0.001). The mean heart rate in the same group and time points was 79.78 ± 15.77, 78.33 ± 13.89, and 78.56 ± 14.43, respectively (*p* = 0.484). Similarly, the systolic and diastolic blood pressure (BP) were 136.56 ± 15.56 over 90.56 ± 10.60, 130.22 ± 20.24 over 84.89 ± 8.62, and 133.11 ± 21.26 over 84.22 ± 9.32 beats per minute (BPM), respectively, (*p* < 0.001 for both measures).

Four eyes of two patients were started on either topical or oral IOP-lowering medications at some point during the study. Four eyes of another two patients had no changes made in therapy during the study.

Three of the thirty PPP eyes (10%) found in two patients had any recorded cataract progression during the study. One patient had cataract surgery in one eye between the week-12 and week-24 visits for worsening posterior subcapsular cataract which was noted after the patient was not using RCI reliably – of note, surgery was performed when active uveitis was not seen on exam but only leakage on UWFA was seen. The other two eyes were in a single patient where one-step worsening cortical changes were noted in both eyes at the week-24 visit that were not present at the week-12 visit; the changes in this patient were not reported to be symptomatic.

##  DISCUSSION

The results of this study suggest that RCI can be effective in the treatment of patients with retinal vasculitis of various etiologies. Our primary outcome of improvement of 50% or more by week 12 was met in half the eyes studied and 65.2% of the eyes of patients by RL method who were able to continue the study medication without deviation until week 12, including an eye with severe vasculitis. In addition, our separate and more traditionally designed scoring method, or RVSS, which was based on tabulation of defined features in various cross-sections of UWFA imaging, yielded percentages of eyes with the primary outcome for improvement in scoring that were similar at 53.3% and 69.6%, respectively at week 12. And while there is no standard accepted method to evaluate retinal vasculitis severity nor response to therapy, the consistency seen with these separately applied methods may demonstrate they both could be viewed as reasonable means of evaluating RV for the purposes of this study.

There was also a statistically significant progressive increase in RL seen in eyes of patients who maintained the study protocol without deviation, despite the number of PPP patients decreasing through the trial period as would be expected in a proof of concept study [Figures 2 and 3; Table 4]. This is an important point to highlight, as it is the aim of the authors to show whether there is significant potential (>25%) for therapeutic benefit with use of this medication for a difficult-to-treat and variably presenting disease process such as retinal vasculitis. Rapid onset of improvement (≤2.9 weeks) was also observed in this study.

Furthermore, 23 of the 30 (76.7%) eyes were able to make it to the week-12 visit without a deviation in study protocol for reason of adverse reaction or need for more therapy before this time point, which we argue is widely agreed upon as a sufficient amount of time to allow any therapeutic measure to show efficacy when evaluating whether response is acceptable to continue the current course or whether a change in therapy is required. It is also notable that almost one third of eyes experienced complete remission of disease by RL score by the end of RCI use in this relatively short study time period of 24 weeks, occurring by a mean of only 17.1 weeks of RCI use. The authors note that a statistically significant increase in vitreous cell was noted between baseline and week 12, however, the difference was minimal and fell below one standard deviation.

Removal of initial use of corticosteroid concomitant with RCI employment also arguably provides the benefit of decreasing risk of potential adverse events with concomitant use of medications having similar side-effect profiles. And while there is no guarantee that patients in this study may have fared better or worse without the introduction of RCI, it is arguable that a direct response to therapy could be a more reliable outcome measure than return in inflammation after corticosteroid therapy is withdrawn.

RCI appeared to be well tolerated, stopped in only one patient because of the side effect attributable to the medication itself, an injection-site reaction. It is worth noting that this type of reaction may possibly be seen more frequently in patients who do not allow the medication to adjust to room temperature prior to withdrawal from the vial and injection (as is intended by the manufacturer). General side effects were minimally seen and tolerated by patients without causation alone for termination of study medication use.

A significant increase in mean weight of almost 5 pounds was seen when comparing the PPP patients at week 24 to their screening visit, but no significant gain was seen in the larger amount of PPP patients at week 12 to their screening visit. This could suggest that weight gain, albeit modest in this case, is a potential side effect of RCI and is more likely to manifest after use for more than three months, which then has implications for any potential long-term use of this medication. The effect of medication on systolic blood pressure, diastolic blood pressure, and heart rate was not significant; however, this is only relatable to short-term employment of RCI.

A study by Aggarwal and colleagues assessed the safety and efficacy of RCI for treatment of 10 patients with polymyositis and dermatomyositis utilizing the same dose and schedule over 24 weeks, however, subjects were not only allowed to concomitantly utilize corticosteroid but also to increase by 10 mg as a rescue and remain in the study as needed.^[18]^ Nonserious adverse events were noted and more plentiful in this group than in our study (22 vs 5) with only half the participants, and serious adverse events were seen including avascular necrosis leading to hip arthroplasty and disseminated zoster with pneumonitis requiring hospitalization. The higher number of side effects including serious side effects such as avascular necrosis might be due to concomitant employment of oral corticosteroid with RCI; however, this hypothesis should be examined with more potent and long-term studies. Interestingly, they saw a similar gain in weight in their patients of approximately 5.72 pounds over 24 weeks, however, the change was not found to be significant. Again, we suspect that higher rates of reactions or adverse events may be found in patients who concurrently use systemic corticosteroids and RCI together, due to the partial mechanism of action in the latter and the similarly described potential side effect profile of each.

No clinically significant increase in IOP measurements was seen over the course of the study – although the mean IOP did statistically increase by 0.28 between baseline and week 12, this was much less than 1 mmHg and fell well within one standard deviation. The mean IOP then fell below the baseline level at week 24 but again this was less than 1-mmHg difference and much less than the standard deviation. Despite the small sample size, these findings over 24 weeks seem to suggest significant or concerning elevation of IOP is uncommon with RCI use.

Retinal vasculitis is well known to be a severe type of ocular inflammation that is often refractory to conservative (or even some aggressive) therapeutic measures. Several case reports and retrospective reviews have been published describing varying efforts to treat noninfectious retinal vasculitis, and any associated ocular inflammatory disease, that had previously been poorly responsive to other forms of therapy. Vallet and colleagues showed anti-tumor necrosis factor (TNF) agents, specifically adalimumab and infliximab, to be effective in treating RV associated with Behçet's disease in 124 patients, with either >50% improvement in RV or some improvement along with significant reduction of systemic corticosteroid seen in 90 and 94.9%, respectively, after six months of therapy; they also noted that infliximab was more often used for patients with severe ocular disease, which included RV.^[19]^ Similarly, Sharma and colleagues reported 88.23% of 60 patients with RV, associated with a large variety of ocular inflammatory conditions, who previously failed therapy with several conventional immunosuppressive medications and were later treated with infliximab after which they were able to attain clinical remission at six months after the initiation of therapy, with 100% achieving remission at 12 months.^[20]^ A case of severe bilateral lupus-associated panuveitis and RV was seen to worsen despite pulse intravenous cyclophosphamide and methylprednisolone and later improve only when rituximab was added to these two medications.^[21]^ There are only two previously published case reports of use of RCI in a patient with intraocular inflammation – a patient with panuveitis and diffuse RV, who had previously failed systemic corticosteroid therapy and intravenous tocilizumab, showing sustained improvement in disease activity in this patient at the same dose used in this study,^[22]^ as well as a series of three patients with bilateral, noninfectious anterior and intermediate uveitis who showed improvement in inflammation, stable vision, and ability to reduce mean systemic steroid without any reportable side effects over 14 months of therapy.^[23]^ These examples highlight the importance of further investigation into newer and possibly more effective means of treating RV, such as RCI, especially considering some patients may fail or have a contraindication or adverse reaction to other known typically effective medications.

To the best of our knowledge, this is the first prospective study of patients with noninfectious retinal vasculitis being treated with RCI. We adapted a simple 15-point scale to assess RV severity score and the clinical response via calculation of improvement (or worsening) of this score, which we feel is a feasible calculation that can easily be made even in a clinical setting. We also employed our own method to evaluate RV activity as well as response to therapy for the purpose of this study, as it is conceivably difficult (and often confusing) to objectively measure levels of a disease process that presents so variably, and there is no widely accepted standardized method in use for this form of ocular inflammation. Clinically, retinal vasculitis is typically treated by observation of overall response to therapy, thus we believe subjective RL method to be easily translatable to practice for those with familiarity for interpreting FA. In finding a similar progression of score and response to therapy via two independent methods, we again feel this suggests that either of these methods could be a valid means of following RV severity and response to therapy.

There are several limitations to this study, which include its open-label status, a limited study period of only 24 weeks, the lack of a control population, varied etiology and severity of disease, concomitant use of other immunomodulatory medications, as well as the use of patients either on or off these other forms of therapy. As stated above, the patient population represented a variable group of disease processes that had retinal vasculitis, as might be expected in a condition or population being studied where the number of patients is few. Criticism could be made to this point of the validity of any conclusions brought forth in this manuscript, however, with the etiology of these processes being at least agreeably noninfectious in nature, the authors expect that these data contain merit in the way of an observed response of a noninfectious inflammatory process to a single given therapy. Lastly, though this study was performed with funding provided by “Mallinckrodt Pharmaceuticals, Bedminster, NJ”, the authors were solely responsible for protocol development, initiation of patient recruitment, administration of study protocol, as well as manuscript production.

In conclusion, given these findings, we therefore recommend that RCI be considered as a potential agent in the treatment of patients with RV at a dose of 80U twice weekly, including patients who have not responded to other types of anti-inflammatory therapy. We also contend that the use of prednisone need not be concurrent while still seeing rapid benefit in this population with tendency toward severe disease activity. These findings should be evaluated with more potent studies with larger sample sizes.

##  Financial Support and Sponsorship

This trial was supported by an investigator-initiated research grant from Mallinckrodt (Staines-upon-Thames, United Kingdom). Mallinckrodt had no role in the design or conduct of this research nor production of this manuscript.

##  Conflicts of Interest

None of the authors have any conflict of interest with the content of this manuscript. ClinicalTrials.gov identifier: NCT03066869

##  Disclosures

i. Dr. C Stephen Foster declares the following:

Consultancies with Aldeyra Therapeutics (Lexington, MA), Allakos (Redwood City, CA), Bausch & Lomb Surgical, Inc. (Rancho Cucamonga, CA), Eyegate Pharma (Waltham, MA), Genentech (South San Francisco, CA), Novartis (Cambridge, MA), and pSivida (Watertown, MA).

Grants or grants pending with Aciont (Salt Lake City, UT), Alcon (Aliso Viejo, CA), Aldeyra Therapeutics (Lexington, MA), Bausch & Lomb (Rochester, NY), Clearside Biomedical (Alpharetta, GA), Dompé pharmaceutical (Milan, Italy), Eyegate Pharma****(Waltham, MA), Mallinckrodt pharmaceuticals (Staines-upon-Thames, UK), Novartis Pharmaceuticals (Cambridge, MA), pSivida (Watertown, MA), and Santen (Osaka, Japan).

Payment for lectures including service on speaking bureaus: Alcon (Aliso Viejo, CA), Allergan (Dublin, Ireland), Mallinckrodt pharmaceuticals (Staines-upon-Thames, UK).

Stock or Stock Options: Eyegate Pharma (Waltham, MA).

ii. Dr. Stephen D. Anesi declares the following:

Consultancies with Santen (Osaka, Japan), Mallinckrodt (Staines-upon-Thames, UK), Allakos (Redwood City, CA), Eyepoint (Watertown, MA), and Takeda (Tokyo, Japan).

Speakerships with AbbVie (Chicago, IL), Mallinckrodt (Staines-upon-Thames, UK), and Eyepoint (Watertown, MA).

iii. Dr. Peter Chang declares the following:

Consultancies with Eyepoint (Watertown, MA) and Alimera (Alpharetta, GA).

Speakerships with AbbVie (Chicago, IL), Mallinckrodt (Staines-upon-Thames, UK), and Eyepoint (Watertown, MA).

All other authors have no proprietary or commercial interest in any materials discussed in this article or additional financial disclosures to declare.

##  Data Availability Statement

The data that support the findings of this study are available from the corresponding author [CSF] upon reasonable request.
